# Association Between Self-Reported Sleep Problems and Cognitive Decline: A Two-Wave Prospective Study

**DOI:** 10.1093/geroni/igaf122.3092

**Published:** 2025-12-31

**Authors:** Fumiko Hamada, Monica Walters, Victor Molinari

**Affiliations:** University of South Florida School of Aging, Tampa, Florida, United States; University of Michigan, Ann Arbor, Michigan, United States; University of South Florida, Tampa, Florida, United States

## Abstract

Sleep is critical to our health, and multiple studies suggest sleep health relates to cognition. However, there are individual differences in sleep problem trends that may influence patterns of cognitive decline. We examined associations between sleep problem trends and cognitive decline among 437 older adults (MageT1=70.3, female=60.0%) from Midlife in the United States (MIDUS) II (Time 1 [T1]:2005-2006) and III (Time 2 [T2]:2013-2017). We created four sleep problem trends from T1-T2 based on self-reported difficulty staying asleep: (1) “no sleep problems” at either time point (n = 259), (2) “developed” sleep problems between T1-T2 (n = 53), (3) “recovered” from T1 sleep problems at T2 (n = 48), and (4) “sustained” sleep problems from T1-T2 (n = 77). Cognition was measured with the Brief Test of Adult Cognition by Telephone. Covariates included age, race/ethnicity, education, and self-reported physical health. Residualized change regressions assessed differences in cognitive decline between groups. Compared to participants with no sleep problems, participants with the other sleep problem trends did not have greater decline in cognition in unadjusted or adjusted models. Regardless of sleep problem trends, all groups experienced significant cognitive decline. While individual differences in sleep problems exist, the findings suggest that sleep problems, whether sustained, developed, or recovered, may not exacerbate cognitive decline. This underscores the importance of considering broader factors influencing cognitive health beyond self-reported sleep difficulties. Further research is needed to explore potential interventions that could help preserve cognitive function in the context of age-related sleep changes.

